# Evaluation of choroidal thickness with OCT in COVID-19 patients with high D-dimer levels

**DOI:** 10.1038/s41598-022-21579-5

**Published:** 2022-10-18

**Authors:** Fatih Cem Gül, Esra Suay Timurkaan

**Affiliations:** 1 Universal Eye Center, Ophthalmology Clinic, Elazig, Turkey; 2 Elazig City Hospital, Elazig, Turkey

**Keywords:** SARS-CoV-2, Eye diseases

## Abstract

To evaluate retinal and choroidal thickness with optical coherence tomography (OCT) to detect retinal and choroidal pathologies in coronavirus disease 2019 (COVID-19) patients with high D-dimer levels. Thirty patients who were hospitalized in the intensive care unit due to severe acute respiratory syndrome coronavirus 2 and whose D-dimer levels were high during this period, who applied to the internal medicine outpatient clinic between 15 and 30 days after discharge, and 30 healthy volunteers with similar age and gender as the control group was included in the study. After full ophthalmological examination, central foveal and choroidal thicknesses were evaluated using optical coherence tomography. Statistical analysis of the study data demonstrated that there was no significant difference between the groups in terms of age or gender (*p* > 0.05). There was also no statistically significant difference between the groups in terms of central foveal thickness, central choroidal thickness, or nasal 500, nasal 1500, temporal 500, or temporal 500 micron distances (*p* > 0.05 for all parameters). Choroidal and retinal vascular thicknesses were not affected in the short term in COVID-19 patients hospitalized in the intensive care unit.

## Introduction

Severe acute respiratory syndrome coronavirus 2 (SARS-CoV-2) is a subtype of the coronavirus family that causes coronavirus disease 2019 (COVID-19) in humans and animals^1^. Affecting millions of people worldwide, COVID-19 can cause a wide spectrum of diseases ranging from asymptomatic disease to life-threatening respiratory distress^2^. Since the beginning of the pandemic, publications on ocular involvement have been increasing. Previously, only anterior segment involvement was well known and virus involvement was thought to be from the anterior surface of the eye. Nowadays, posterior segment involvement due to direct viral invasion, hematologic spread and systemic inflammation has gained importance.

Since SARS-CoV-2 can bind to angiotensin-converting enzyme-2 (ACE-2) in the respiratory system, eye, esophagus, ileum, liver, heart, kidney, testis, and endothelium, it has the potential to cause disease^3^. COVID-19 can cause hepatic, enteric, neurologic, and nephrologic disorders. ACE-2 is a receptor located on the plasma membrane of tissue epitheliums^3^. ACE-2 receptors can be found in human eye tissues such as the conjunctiva, cornea, ciliary body, choroid, Müller cells, ganglion cells, photoreceptors, and retinal vascular endothelial cells^3,4^. In addition to ACE-2 receptors, CD147 transmembrane glycoprotein, which is numerously present in many cells of the body, including the retina, is an important structure for SARS-Cov-2 to enter tissues^5,6^. It is also thought that SARS-Cov-2 crosses the blood retinal barrier into leukocytes^7^. SARS-CoV-2 was a pathogen that could cause a multisystem inflammatory syndrome. Ocular tissues, like any other part of the body, can be affected. SARS-CoV-1 and SARS-CoV-2 proteins were detected in the samples taken from the tear film layer^8,9^. Potential involvement of the virus in the internal/external retinal layers and vitreous has been reported^10^. In addition, in the postmortem examination of 14 patients who died due to COVID-19, SARS-CoV-2 ribonucleic acid was detected in the retina of 3 of the patients^11^. These evidence supports the presence of the virus in ocular tissues.

SARS-CoV-2 causes thrombosis, microangiopathy, and angiogenesis-related damage to the vascular endothelium^12^. This damage may occur due to direct virus invasion as well as activation of the complement system^13^. The choroid, which forms the posterior part of the uveal tract, provides oxygenation and nutrition to the outer retina and photoreceptors^14^. In addition, the choroid is responsible for the blood supply of the retinal outer segment, retinal pigment epithelium, and the prelaminar part of the optic disc^15^. As a result, choroid abnormalities play a role in the pathogenesis of a variety of retinal diseases. The choroid, which is the vascular layer of the eye, is one of the areas of the body where blood flow is most intense. Choroidal thickness may be affected by age, refractive error, axial length, diseases such as Alzheimer's or glaucoma^16–18^. Since COVID-19 is known to affect microvascular circulation in many organs, it can be expected to cause an effect in a structure with high blood flow and microvascular network such as the choroid. In addition, it has been reported that choroidal inflammation can develop in multisystem inflammatory disease due to COVID-19, while there is no evidence of ocular involvement.

In recent years, choroidal thickness measurements have also become important for eye diseases. Choroidal and retinal thickness measurements have been reported to be potential structural markers of inflammation in previous studies^19,20^. Choroidal thickness can be measured in vivo with USG, MRI, and OCT. OCT is a noninvasive method that provides cross‑sectional imaging of the retina and choroid and has been used to evaluate choroidal thickness with acceptable reproducibility and sensitivity^21^.

D-dimer is a product of fibrin degradation and is released into the circulation when the coagulum is dissolved by fibrinolysis^22^. It has two D fragments of the fibrin protein attached by a cross-link^23^. It is an important laboratory parameter that shows coagulation in diseases such as COVID-19 that progress to coagulation. Retinal vascular thromboembolic events in COVID-19 patients have been reported to be associated with the total embolic load of the patients^24^. Elevated D-dimer levels are an important laboratory parameter related to thromboembolic events.

In our study, we aim to detect possible pathologies that have developed in choroidal vessels, with OCT, a non-invasive diagnostic method in patients with high levels of D-dimer, a coagulation marker, in COVID-19 disease that can cause a tendency to coagulation.

## Material and methods

This prospective study was approved by the Institutional Review Board of Firat University, and all procedures were applied in compliance with the Declaration of Helsinki. Written and signed approvals were obtained from all participants. All methods were performed in accordance with the relevant guidelines and regulations by including a statement.

Our study included patients who were hospitalized in the intensive care unit, had their COVID-19 treatment completed, and came to our internal medicine outpatient clinic for follow-up (15–30 days) after discharge. COVID-19 information of the patients was analyzed retrospectively. Patients' diagnoses of COVID-19 were confirmed by both thorax computed tomography (CT) and Polymerase Chain Reaction (PCR) tests. The D-dimer level (serum) of the patients was above the reference range (above 0–0.5 µg/mL in our hospital). The patients' FiO2 levels were in the range of 0.8–0.9 mmHg, their oxygen saturation was between 85 and 90%, and patients did not require intubation or mechanical ventilator. Patients were given a standardized COVID-19 treatment that included a 2 × 1600 mg favipiravir loading dose, a 5-day maintenance dosage of 2 × 600 mg favipiravir, 1 mg/kg/day methylprednisolone, enoxaparin sodium 2 × 4000 IU, and pantoprazole 2 × 40 mg. Patients who had previously used antithrombotic and/or antiaggregant therapy, had a history of cerebrovascular events, cardiovascular events, thrombosis, embolism, and were diagnosed with malignancy were not included in the study. Best corrected visual acuity, intraocular pressure values, and detailed anterior and posterior segment examinations with slit lamps were performed. All patients were asymptomatic and no positive findings were found in ophthalmological examinations.

All Central fovea and choroidal thicknesses were measured using OCT (Canon OCT-HS 100). The Canon OCT-HS100 device has a scanning speed of 70,000 A-scans/sec with an axial resolution of 3 μm and a scanning depth of 2 mm. The choroid mode, which gives high quality images, was used during the measurements. Choroidal thickness was measured vertically between the outer border of the hyperreflective line corresponding to the RPE and the inner surface of the sclera. Choroidal thickness was measured from the fovea and at 500 and 1500 µm nasally and temporally to the fovea. All measurements were performed by the same experienced blinded individual.

Patients with a known eye disease, systemic disease that can involve the retina (such as diabetes, hypertension), ocular pathologies (such as age-related macular degeneration, uveitis), glaucoma, retinal axial length < 20 mm or > 24 mm, using drugs that may be toxic to the retina, and those who underwent ocular surgery were excluded from the study. In addition myopia and hyperopia were defined as the spherical equivalent of subjective refraction ≤ − 0.5 D and > + 0.5 D, respectively and the patients above these diopters were excluded from the study.

### Statistical analysis

Statistical Package for the Social Sciences (SPSS) version 22.0 software (SPSS Inc., Chicago, IL, USA) was used for statistical analysis. The normality of the distribution of the variables in the statistical evaluation was examined with the Kolmogorov–Smirnov test. Wilcoxon Signed Rank Test, a nonparametric test, was used as there is no normal distribution between the groups. Descriptive analyzes are given with percentage, mean, standard deviation, and median. The means were given together with the standard deviation (Mean ± SD), p < 0.05 was evaluated as statistical significance.

### Ethical approval

The study was approved by the Ethics Committee of the Firat University, Elazig, Turkey (2021/08-35).

### Patient’s constent

The informed and written consents of the patients were signed by the patients before the beginning of the study.

## Results

**Figure 1:**
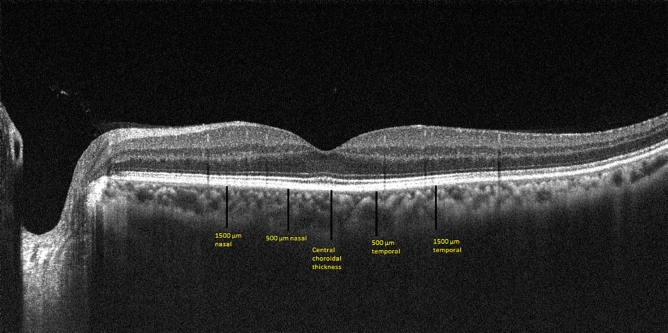
Choroidal thickness measurements on optical coherence tomography (OCT). Vertical lines are drawn from retinal pigment epithelium to sclera.

Thirty patients (Group 1), 14 men and 16 women with a mean age of 47.38 ± 7.3 years who had COVID-19 15–30 days before, and 14 men and 16 women in a healthy control group (Group 2) with a mean age of 47.72 ± 6.23 years were included in the study. There was no statistically significant difference between the groups in terms of age (p = 0.62). The mean central foveal thickness was 251.76 ± 9.71 μm in group 1 and 250.72 ± 7.93 μm in group 2 (p = 0.76). The mean central choroidal thickness was 361.03 ± 17.97 μm in group 1 and 361.52 ± 20.01 μm in group 2 (p = 0.78). In measurements taken from the 500 micron nasal of the macula, the choroidal thickness in group 1 was 301.14 ± 24.64 µm, while it was 297.24 ± 9.67 µm in group 2 (p = 0.90). In the measurements taken 500 microns temporally of the macula, the choroidal thickness was 295.03 ± 9.0 μm in group 1, while it was 296.10 ± 6.13 μm in group 2 (p = 0.29). In measurements taken from the 1500 micron nasal of the macula, the choroidal thickness was 268.38 ± 21.41 μm in group 1, while it was 268.38 ± 20.52 μm in group 2 (p = 0.98). In the measurements taken from the 1500 micron temporal of the macula, the choroidal thickness was 268.24 ± 16.13 μm in group 1, while it was 268.83 ± 14.22 μm in group 2 (p = 0.69). There was no statistically significant difference between group 1 and group 2 in choroidal and macular thickness measurements (Table 1), (Fig. 1).

**Table 1:** Age, gender, central macular thickness, coroidal thickness and p value of the study groups. (M: Male, F: Famale, CFT: Central Foveal Thicknesss, CT: Coroidal thickness).

	Group 1	Group 2	*p* value
Age	47.38 ± 7.3	47.72 ± 6.23	0.62
Gender	14 M/16 F	14 M/16 F	1.00
CFT (μm)	251.76 ± 9.71	250.72 ± 7.93	0.76
Central CT (μm)	361.03 ± 17.97	361.52 ± 20.01	0.78
Nasal 500 CT (μm)	301.14 ± 24.64	297.24 ± 9.67	0.90
Nasal 1500 CT (μm)	268.38 ± 21.41	268.38 ± 20.52	0.98
Temporal 500 CT (μm)	295.03 ± 9.0	296.10 ± 6.13	0.29
Temporal 1500 CT (μm)	268.24 ± 16.13	268.83 ± 14.22	0.69

The D-dimer levels of the Group 1 was 5.131 ± 2.394 µg/mL in the intensive care unit and 0.335 ± 0.174 µg/mL in the post-covid period (15–30 days after recovery). There was a statistically significant difference between D-dimer levels (p < 00.1). In Group 2, D-dimer levels were 0.303 ± 0.157 µg/mL at the beginning of the study and 0.307 ± 0.102 µg/mL in the post-covid period. There was no statistically significant difference between D-dimer levels (p = 0.915). There was a statistically significant difference in D-dimer levels between Group 1 and Group 2 at the beginning of the study (p < 0.001). In the post-covid period, there was no significant difference in D-dimer levels between the groups. (p = 0.451) (Table 2).

**Table 2:** D-dimer levels of the groups.

D-Dimer (µg/mL)	Group 1	Group 2	*p* value
At the beginning of the study	5.131 ± 2.394	0.303 ± 0.157	< 0.001
Post-COVID period	0.335 ± 0.174	0.307 ± 0.102	0.451
*p* value	< 0.001	0.915	

## Discussion

It is not clear how COVID-19 affects the eye and for how long. It is thought that COVID-19 infection enters the eye by several mechanisms. Virus invasion can be in the form of direct virus invasion from the cornea and conjunctiva, hematogenic spread due to disruption of the blood–brain barrier, and neuronal retrograde transport^25^. It is thought that its effects in the tissue are due to the effects caused by virus invasion on the vessel wall, activation of the complement system, or systemic disease caused by virus^26^. In addition It has been reported that COVID-19 causes damage to the vascular endothelial glycocalyx, resulting in systemic inflammatory microvascular endotheliopathy^27^. This pathology caused by COVID-19 in the vascular tissue creates a tendency to coagulation. The choroid may therefore be an important target organ.

It has been reported in the literature that D-dimer levels are correlated with the severity of COVID-19, and higher D-dimer levels are detected in patients with severe disease than those with milder disease^26^. In a study by Huang et al., D-dimer levels were found to be elevated in 260 out of 560 COVID-19 patients^27^. Wang et al. reported that patients with D dimer levels of 2.5 times or greater had a higher risk of mortality^28^. D-dimer is a laboratory parameter that indicates increased coagulation in the body. Intravascular coagulation resulting in end-organ damage and embolism tendency secondary to hypoxia increased in COVID-19 disease^29^. The incidence of thromboembolic events in patients with acute respiratory distress syndrome (ARDS) due to COVID-19 has increased compared to other causes of ARDS^30^**.** The risk of thromboembolic complications due to COVID-19 in intensive care patients has been reported to be 31.7%^31^. In another study, it was reported that 30% of patients with COVID-19 had venous or arterial thromboembolic events^32^. In COVID-19, dark spots showing hypoperfusion in the choriocapillaris have been detected in OCT angiography. This finding was especially detected in patients with high D-dimer levels^33^. Therefore, D-dimer levels of COVID-19 patients are particularly important and there is a risk of pathological changes due to increased coagulation in tissues with high blood supply, such as the choroid.

Retinal lesions in COVID-19 patients were first reported by Marinho et al. as asymptomatic cotton wool spots and microhemorrhage in 4 of 12 COVID-19 patients^34^. Paracentral acute middle maculopathy affecting deep retinal vessels in the inner nuclear layer, and acute macular neuroretinopathy characterized by microangiopathy affecting the outer nuclear layer and outer plexiform layer are also reported in COVID-19 patients^35,36^. It has also been reported that retinal vascular occlusion can be seen with COVID-19 infection^37,38^. In their study, Invernizzi et al. found that retinal hemorrhage was observed in 9.25%, soft exudate in 7.4%, dilated veins in 27.7% and tortuous vessels in 12.9% in fundus examinations of COVID-19 patients. They thought that these changes were due to inflammation, hypoxia, and increased carbon dioxide levels^39^. In the study of Yaser et al., choroidal leakage and staining were detected in 71% of the patients in COVID-19. It has been reported that this leakage may be due to direct Sars-Cov2 damage in the glycocalyx^33^. In our study, ophthalmological examination findings were not included at the early period of covid disease due to risk of contagion and poor general health condition. In the ophthalmological examination performed at the post-covid period, we did not detect any pathological findings in the fundus. Since we evaluated the patients at the post-covid period, possible lesions may have healed.

Chorioretinitis is strongly associated with systemic or local infectious and inflammatory conditions. Because the choroid is a densely vascularized tissue, it is sensitive to systemic diseases, especially those that affect the blood vessels. Glaucoma, retinitis pigmentosa, degenerative myopia, and age related macular degeneration (AMD) all result in decreased choroidal blood flow. Histological examination of AMD revealed edema, fibrosis, and cellular infiltration in the choroid^40^. It has been reported in the literature that COVID-19 causes choroidal involvement. Ortiz Seller et al. reported a case of inflammatory chorioretinitis, which they thought belonged to the spectrum of white dot syndrome^41^. In addition, two cases of panuveitis considered related to COVID-19 were reported, both of which healed after systemic and local therapy^42,43^. Reactivation of serpiginous choroiditis due to the immune response induced by COVID-19 has also been reported^44^.

Systemic diseases have been shown to affect retinal and choroidal blood flow. In the study of Hayreh et al., it was shown that hypercapnia causes vasodilation of the choroidal vessels and affects choroidal perfusion^45^. Choroidal thickening was found in Vogt-Koyanagi-Harada and Behçet's disease^46,47^. The choroidal thickness in these diseases normalized after the treatment. In addition, Agarwal et al. found that choroidal thickness increased in HIV patients, especially in those with HIV microangiopathy, compared to healthy controls^48^. There are several studies with different results regarding choroidal involvement in COVID-19 patients. Türker et al. reported that an increase in choroidal perfusion with OCT angio in COVID-19 patients. They linked it to hypoxia-induced vasodilation in the choroidal vessels, and an increase in flow in the choroid, which is not autoregulated, such as the retina, as a result of systemic inflammation^1^. Firat et al. reported that the choroidal thickness of patients with a relatively mild course who had COVID-19 but were not hospitalized, and no significant difference was found in the choroidal thickness compared to the control group^49^. In the study of Hepokur et al., choroidal thickness was found to decrease in COVID-19 during the early post-infectious period and to normalize during the late post-infectious period^50^. However, this study was performed in the early period of the COVID-19 pandemic and there is no anticoagulant treatment in the treatment protocol. The reason why we could not find any difference between choroidal thicknesses in our study can be interpreted as the fact that the patients received anticoagulant treatment and that the period in which they were examined was the post-infectious period.

In previous studies, it is not clear whether the lesions detected in the retina and choroid are directly due to COVID-19 or are related to the previous diseases of the patients. As we mentioned above, there are studies in which retinal or choroidal lesions were not detected despite severe COVID-19 infection. We also did not detect any difference in choroidal thickness in our study. In addition, cases with choroidal involvement were usually identified as a single case or not defined in large series. For this reason, we suggest that studies with larger patient participation and especially during the active period of COVID-19 are important in demonstrating the changes that COVID-19 may cause in retina and choroidal structures.

In our study, our patients consisted of patients with intense oxygen requirements, followed in the intensive care unit, and high D-dimer levels showing increased coagulation. Therefore, it is important in terms of showing the possible effects of severe COVID-19 infection on the choroid. There was no statistical difference between the choroidal and retinal thicknesses of the patients in our study compared to the control group. The fact that we did not detect any difference in retinal and choroidal thickness in our study may be due to the anticoagulant treatment applied to the patients, relatively young age, the absence of associated systemic diseases, and the absence of short-term sequelae in other systems related to COVID-19.

There are also some limitations of our study. First of all, the patients received anticoagulant treatment in the intensive care unit. Second, our data does not show the long-term results of the patients. In addition, OCT cannot detect all choroidal vascular changes.

## Conclusion

Choroidal and retinal vascular thicknesses were not affected in the short post-COVID period in COVID-19 patients hospitalized in the intensive care unit.

## Data Availability

The datasets used and/or analysed during the current study available from the corresponding author on request.
